# An enhanced hypergraph CNN with adaptive focal loss for automated ECG heartbeat classification

**DOI:** 10.1038/s41598-026-45518-w

**Published:** 2026-05-27

**Authors:** Akash Vijayan, Suchetha Manikandan, Deepak Joshi

**Affiliations:** 1 Centre for Healthcare Advancement, Innovation and Research, Vellore Institute of Technology, Chennai Campus, Chennai, TN 600127 India; 2 Centre for Biomedical Engineering, Indian Institute of Technology, Delhi, 110016 India

**Keywords:** Deep learning, Electrocardiogram (ECG), Cardiovascular disease diagnosis, Convolutional neural networks, Hypergraph neural networks, Arrhythmia detection, Cardiology, Computational biology and bioinformatics, Diseases, Engineering, Health care, Mathematics and computing

## Abstract

Deep learning techniques have shown significant promise for the automated diagnosis of CVD using ECG analysis. Nevertheless, several critical challenges persist with current approaches: severe class imbalance, intricate temporal dependencies, and poor modeling of inter-beat relationships ultimately limit clinical applicability. This work presents a novel hybrid deep learning framework that combines a CNN for time-domain feature extraction, k-nearest neighbor-based hypergraph construction with cosine similarity to model inter-beat dependencies, and a residual-connection-enhanced hypergraph neural network (EHGNN) for robust classification. To address class imbalance, focal loss is implemented with adaptive class weighting. For evaluation, our model was tested on two benchmark datasets: the MIT-BIH Arrhythmia Database and the St. Petersburg Institute of Cardiological Technics (INCART) 12-lead Arrhythmia Database. In the case of the MITBIH dataset, a classification accuracy of 98.66% was achieved using the AAMI five-class classification system, whereas the results indicated a three-class arrhythmia detection accuracy of 95.46% for the INCART database. The model demonstrated consistent performance on two separate data sets, thus emphasizing its ability to generalize well. These results confirm that the proposed framework effectively addresses class imbalance through focal loss and SMOTE, temporal dependencies through CNN-based feature extraction, and inter-beat relationships through EHGNN, demonstrating great potential for clinical diagnostic systems by capturing complex heartbeat patterns that are otherwise overlooked by standard models

## Introduction

**Fig. 1:**
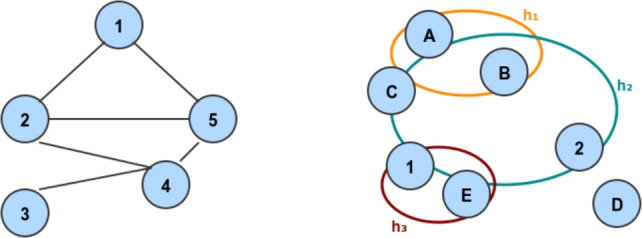
Comparison of (**a**) a conventional pairwise graph and (**b**) a hypergraph with multi-vertex hyperedges.

As the World Health Organization confirms, cardiovascular diseases are at the top of the list of global fatalities, accounting for 31% of all fatalities and 17.9 million annual deaths worldwide^1^. Among the wide range of cardiovascular diseases, cardiac arrhythmias are one of the specially noticed conditions because of the sudden occurrence and potentially lethal results, making the diagnostic and treatment process extremely challenging. In order to enhance success rates and decrease mortality rates, early and correct diagnosis of arrhythmias is the key. To date, the ECG is the gold standard method of diagnosis because of the high accuracy of the cardiac activation timing that is of prime importance in the precise diagnosis of cardiac rhythms^2,3^. Experienced cardiologists can also use their naked eye to notice subtle features in an ECG signal such as variations in the ST segment, P wave characteristics, and the QRS complexity in order to infer the possibility of certain Arrhythmias. However, manual analyses are rather subjective, consuming, as well as prone to inter-observer variability. Excess fatigue, inter-observer variability, and the subjective nature of such analyses can also influence their accuracy. This has led to the development of automated ECG signal processing systems for efficient diagnosis. In the early stages of automation, most approaches relied on manually designed feature extraction combined with traditional machine learning techniques. These systems typically extracted morphological and temporal features, such as QRS complex duration, RR intervals, P-wave amplitude, and ST-segment deviation^4–6^.In some scenarios, people also used frequency-domain features from Fourier transforms or wavelet analyses. All these methods may reach decent results on balanced datasets, but they mostly struggle with minority classes, have a limited generalization, and are heavily dependent on domain expertise to craft the right features. With the introduction of deep learning, the automated ECG analysis changed drastically, In an end-to-end configuration, models can now learn the representation of features directly from raw signals without the extraction of hand-engineered features. Among the various architectures, CNNs are unparalleled for ECG classification, especially adept at picking out local spatial patterns and unique shapes in heart wave forms. However, standard CNNs process each fixed-length segment independently and lack any capability to capture long-range temporal dependencies that characterize sequential cardiac events across multiple heartbeats^7^. RNNs and LSTM networks have been adapted to capture sequential dependencies in ECG signals by incorporating them to compensate for the limitations of temporal modeling^8–10^. The hybrid CNN–LSTM architectures have evinced great success by effectively fusing spatial feature extraction and temporal sequence modeling^11^. However, all these methods are still susceptible to vanishing gradients during training and present computational challenges as ECG recording lengths increase. Very recently, transformer-based architectures with inherent self-attention mechanisms have been explored for modeling long-range dependencies without recurrence. Though these models have achieved great success in many other domains, they express limited effectiveness in modeling complex, multi-scale, and hierarchical relationships that are naturally involved with ECG signals, which consist of intricate interactions among different cardiac events as well as across multiple leads^12,13^. Graph Neural Networks (GNNs) have recently gained attention in ECG analysis because of their inherent ability to model relationships among cardiac events as graph-structured data^14^. By representing ECG beats as and their relationships as edges, GNNs can capture spatiotemporal dependencies that are challenging for conventional network architectures. However, traditional GNNs are fundamentally limited to modeling pairwise relationships between, proving insufficient for capturing the high-order dependencies present in multi-lead ECG recordings and inter-beat dynamics. Hypergraph Neural Networks (HGNNs) extend this representation to hypergraphs, where a single hyperedge can connect multiple simultaneously as shown on Fig. 1, enabling the modeling of complex many-to-many relationships^15,16^. This capability is especially relevant in ECG analysis, where arrhythmia patterns frequently involve coordinated changes across several leads and consecutive heartbeats.

In this work, we propose a hybrid deep learning framework that combines CNNs for capturing local features with HGNNs for capturing high-order relationships. We balance the relative strengths of the two architectures while carefully tuning the architecture and training strategy to amend their respective limitations. Our model segments data using fixed-size windows aligned with anchor annotations, providing improved reliability in the beat extraction process. We further employ physiology-inspired data augmentation to enhance generalization and apply focal loss with adaptive class weighting to address the pronounced class imbalance.

**The main contributions of this work are as follows:**

- We present a new hybrid architecture that integrates CNN-based temporal feature extraction with a k-nearest neighbor hypergraph construction using cosine similarity, enabling explicit modeling of inter-beat relational dependencies that are inherently overlooked by standard sequential and convolutional models.
- We developed an Enhanced Hypergraph Neural Network (EHGNN) with residual connections across hypergraph convolutional layers and batch normalization, yielding significantly improved gradient flow, training stability, and convergence speed compared with standard hypergraph architectures.
- We propose a comprehensive data processing pipeline that includes annotation-anchored segmentation, physiologically constrained augmentation, and focal loss with class-specific weighting to effectively address the serious class imbalance problem in clinical arrhythmia datasets.
- Extensive experimental validation is carried out on two benchmark datasets with distinct characteristics: the MIT-BIH Arrhythmia Database^17^ achieving 98.66% accuracy for five-class AAMI classification) and the INCART 12-lead Arrhythmia Database (achieving 95.62% accuracy for three-class classification), demonstrating superior performance, robustness, and generalizability across diverse recording conditions^18^.

## Methodology

### Overall architecture

**Fig. 2:**
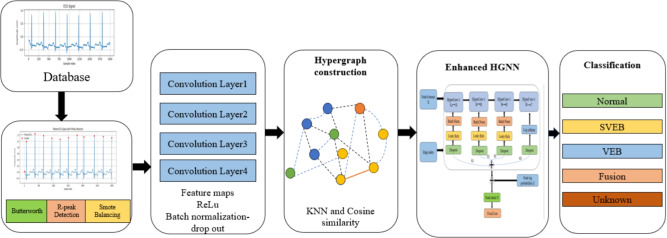
Overview of the proposed multi-stage ECG arrhythmia classification framework.

The overall architecture of the model proposed is shown in Fig. 2. The model follows a multistage pipeline architecture that integrates CNNs with hypergraph neural networks in order to solve the complex tasks involved in automated cardiac rhythm analysis. We initialize by preprocessing the original ECG signals through bandpass filtering (0.5–50.5 Hz) to eliminate baseline wander, powerline interference, and high-frequency noise using a 4th-order Butterworth filter. In our CNN model, we adopt sequential convolutional layers with increasing filter depth to extract morphological features from single beats. Then we construct the hypergraphs by k-nearest neighbors based on cosine similarity of CNN features that present the morphological relationship across beats. Finally, an enhanced HGNN with residual connections along with focal loss performs classification through specialized hypergraph convolutions to predict arrhythmia predictions.

### Feature extraction via convolutional neural networks

A CNN-based feature extractor is employed to automatically learn discriminative representations from preprocessed ECG segments. The architecture is designed to handle recordings with different sampling rates and extract hierarchical morphological features suitable for both the MIT-BIH Arrhythmia Database (360 Hz) and the INCART Database (257 Hz). All ECG segments are standardized to 256 samples via annotation-anchored segmentation, corresponding to approximately 0.71 s for MIT-BIH and 0.99 s for INCART^19^. This ensures consistent temporal coverage of complete cardiac cycles, including the P-wave, QRS complex, and T-wave.

#### Convolutional feature extraction

**Fig. 3:**
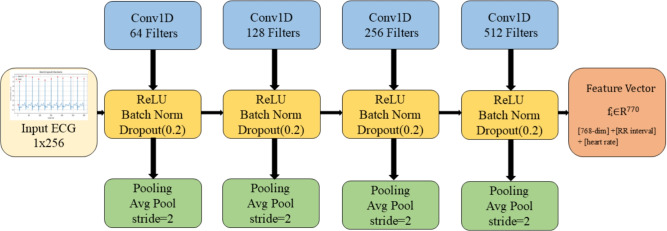
Proposed CNN architecture for ECG beat classification.

The CNN architecture consists of four successive one-dimensional convolutional blocks with progressively increasing filter depths as shown in Fig. 3, enabling capture of multi-scale cardiac morphology. The first layer has 64 filters of size *7 × 7* to capture the shape of low-level evidence such as QRS boundaries, the presence of the P-wave, the beginning of the T-wave, baseline shifts, and peculiarities of high-frequency noise. Its wide receptive field allows it to capture entire waveform structures. The second layer increases complexity with 128 filters and a *5 × 5* kernel, focusing on mid-level, decision-making features, including details of the ST segment (rising or falling shape), polarity and height of the T-wave, evidence from the PR interval, layout of the QT interval, and relationships among wave timings. These features are critical for distinguishing S/NR from other rhythm groups. The third layer, comprising 256 filters with a *3 × 3* kernel, targets fine-grained morphologies such as QRS notches and fragmentation, minute displacements in the P-wave, the presence of U-waves, evidence of premature beat morphologies, and rhythm-specific oscillations such as baseline wobble in atrial fibrillation. The fourth layer employs a set of 512 filters with a 3x3 kernel to detect high-level abstract patterns indicative of complex arrhythmia patterns. It can detect beat-to-beat morphology patterns in beats that correspond to compensatory pauses or fusion beats in addition to detecting patterns at a higher level over multiple beats. Each convolutional block consists of batch normalization to improve convergence during training, ReLU activation to introduce non linearity, and a dropout value of 0.2 to control overfitting during training. Following the sequence of convolutional layers is a hierarchical averaging pooling layer with a pool size of 4x1 and a stride value of 2 to reduce the size of samples along the time axis from 256 samples to a value around 46 samples in two steps. Average pooling is preferred over max pooling to achieve proper morphology rather than minute details that may coincide with isolated spikes of noise in a real-world scenario like a medical environment for a Holter monitor. The feature map is subsequently flattened to a column vector to obtain a feature value from a densely connected layer with a hidden size of 768 to further compute low-dimensional mappings that preserve all levels of morphology features in a compact manner. To incorporate features indicative of rhythms in the input signal, two dimensions comprising feature values of R-R interval and instantaneous heart rates are appended to a densely connected feature map to generate a feature map of a size of 770 features that further form a hypergraph. Let $x_i \in \mathbb {R}^{256}$ denote the *i*-th preprocessed ECG segment and $\theta _{\text {CNN}}$ represent all trainable CNN parameters (convolutional weights, biases, and normalization parameters); the feature extraction process is expressed as $f_i = \textrm{Dense}(\textrm{Flatten}(\textrm{Pool}(\textrm{CNN}(x_i;\theta _{\text {CNN}}))))$, where $f_i \in \mathbb {R}^{768}$ is the learned morphological feature vector. After concatenation with RR-interval and heart-rate information, the final representation becomes 770-dimensional. The CNN architecture was implemented in TensorFlow/Keras and kept identical across both databases, demonstrating strong generalizability to different sampling rates and heterogeneous recording conditions without requiring dataset-specific architectural modifications.The CNN feature extractor operates entirely independently of the hypergraph structure. Each ECG beat is processed in isolation through the convolutional blocks, with no graph connectivity involved at this stage. The hypergraph is constructed exclusively in the subsequent stage using the *k*-nearest neighbour (*k*NN) algorithm applied to the CNN-extracted feature vectors, as described in Section "Hypergraph construction via *k*-nearest neighbors".

## Hypergraph neural network

### Hypergraph formulation

Hypergraph *H = (V, E)* is generated based on a vertex set $V = \{v_1, v_2, \ldots , v_n\}$, where each vertex represents an individual heartbeat signal, and an edge set $E = \{e_1, e_2, \ldots , e_m\}$ involving hyperedges. The distinguishing characteristic of a hypergraph, different from that of a traditional graph, is that the hyperedge $e_i$ may involve any number of vertices, not necessarily pairs. This characteristic makes the hypergraph more suitable for modeling the higher-order relationships that exist among the heartbeats within the ECG signal^20^. In the proposed method for classifying heartbeats, a vertex $v_i$ represents a single ECG heartbeat, and a hyperedge $e_j$ represents multi-beat morphological features among multiple heartbeats. The representation of hyperedges is highly effective for modeling the complex arrhythmic patterns that evolve over a few consecutive heartbeats. For example, bigeminy, which represents alternate occurrences of normal and premature heartbeats, does not form a pairwise relationship suitable for a graph representation, which can easily be represented using hyperedges connecting every alternate heartbeat. Similarly, the irregular and non-pairwise dependencies observed in atrial fibrillation rhythms are more accurately captured through hyperedge-based representations. Let $F = \{f_1, f_2, \ldots , f_n\}$ denote the set of feature vectors extracted using the CNN, where each feature vector $f_i \in \mathbb {R}^{770}$ encodes the morphological and temporal characteristics of the *i*-th heartbeat, comprising 768 features from the CNN dense layer and two additional RR-interval-based features. Next, a weighted hypergraph is developed.

$$\mathscr {H} = (\mathscr {V}, \mathscr {E}, w),$$

where $w: \mathscr {E} \rightarrow \mathbb {R}^{+}$ assigns a weight to each hyperedge representing the strength or reliability of the multi-way similarity relationship.

The feature extraction operation is written formally as:

1 $$\begin{aligned} f_i = \text {CNN}(x_i; \theta _{\text {CNN}}), \end{aligned}$$

where $x_i \in \mathbb {R}^{T}$ is the preprocessed ECG segment of length *T = 256*, $\theta _{\text {CNN}}$ denotes the full set of trainable CNN parameters, and $f_i \in \mathbb {R}^{d}$ is the resulting representation.

#### Cosine similarity distance metric

Cosine similarity is used to measure morphological similarity between heartbeat feature vectors. It offers several advantages:

1. **Scale invariance:** ECG amplitudes vary widely across patients and recording conditions. Cosine similarity emphasizes waveform shape rather than magnitude.
2. **High-dimensional robustness:** Unlike Euclidean distance, cosine similarity remains discriminative in high dimensions by focusing on angular relationships.
3. **Morphological focus:** Diagnostic ECG interpretation relies primarily on waveform shape, which cosine similarity captures effectively.

The cosine similarity between $f_i$ and $f_j$ is defined as:

2 $$\begin{aligned} \text {sim}(f_i, f_j) = \frac{f_i \cdot f_j}{\Vert f_i\Vert _2 \, \Vert f_j\Vert _2} = \frac{\sum _{k=1}^{d} f_i^{(k)} f_j^{(k)}}{\sqrt{\sum _{k=1}^{d} (f_i^{(k)})^2} \; \sqrt{\sum _{k=1}^{d} (f_j^{(k)})^2}}. \end{aligned}$$

The corresponding cosine distance is:

3 $$\begin{aligned} d(f_i, f_j) = 1 - \text {sim}(f_i, f_j), \end{aligned}$$

with $d(f_i, f_j) \in [0,2]$. Small values indicate strong morphological similarity (e.g., consecutive normal beats), whereas large values indicate substantial differences (e.g., normal vs. PVC). Cosine similarity was selected as it remains discriminative in high-dimensional spaces, which is particularly important for ECG signals where amplitude varies across patients while waveform shape remains the primary diagnostic indicator^21,22^. Although the nominal feature dimensionality is 770, the CNN-learned representations have substantially lower intrinsic dimensionality, as evidenced by the clear class separability under t-SNE projection in Fig. 8.

### Hypergraph construction via *k*-nearest neighbors

To construct hyper edges, a *K*-nearest neighbor (k-NN) strategy is applied in the feature space using cosine distance.The *K*NN approach was chosen for hypergraph construction due to its *k × n* directed edges, yielding a sparse and computationally efficient structure and due to its ability to directly connect each beats to its k most similar neighbors in CNN feature space^23–25^. For each vertex $v_i$, its *k* most morphologically similar neighbors form the neighborhood:

4 $$\begin{aligned} \mathscr {N}_k(v_i) = \{ v_j \;|\; d(f_i, f_j) \le d_k^{(i)} \}, \end{aligned}$$

where $d_k^{(i)}$ is the distance to the *k*-th nearest neighbor of $v_i$. For each vertex $v_i$, we form a hyperedge:

**Algorithm 1:**
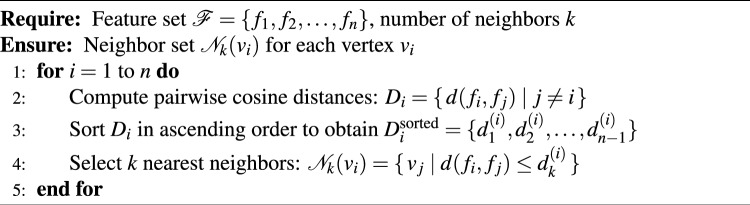
k-NN based hyperedge construction.

5 $$\begin{aligned} e_i = \{v_i\} \cup \mathscr {N}_k(v_i). \end{aligned}$$

Because *k = 5* is determined to be optimal, every hyperedge is connected to exactly six vertices. This is a reflection of the finding that arrhythmia patterns tend to occur in 3 to 7 cycles of the heartbeat^26,27^.In order to empirically validate this decision, a sensitivity analysis over the set of $k \in \{3, 5, 7, 10, 15\}$ was carried out, assessing both the hypergraph structural properties as well as the classification accuracy achieved. The number of nodes and hyperedges is constant across all configurations at 113,883, corresponding to the total number of beats in the MIT-BIH dataset. As depicted in Table 1, the best accuracy rate of *98.66%* and the highest macro F1-score of 0.9397 are obtained when using *k = 5*, thus supporting the decision to connect each beat to its five most morphologically similar neighbours. This choice captures the optimal contextual relationship between beats for accurate arrhythmia classification. While a smaller value of *k = 3* yields sparser graphs that fail to adequately capture morphological contextual relationships, a larger value such as *k \ge 7* produces noisier graphs by connecting beats across dissimilar morphological classes. This negatively impacts minority class discrimination, as evidenced by the lower macro F1-scores obtained.

**Table 1:** Effect of *k* on hypergraph structure and classification performance.

*k*	Hyperedge Size	Directed Edges	Graph Density	Accuracy (%)	Macro F1
3	4	341,649	0.00002634	98.14	0.9281
**5**	**6**	**569,415**	**0.00004391**	**98.66**	**0.9397**
7	8	797,181	0.00006147	98.20	0.9197
10	11	1,138,830	0.00008781	98.55	0.9359
15	16	1,708,245	0.00013172	98.49	0.9327

#### Incidence matrix representation

We represent the hypergraph using an incidence matrix $H \in \mathbb {R}^{n \times m}$, where

A formula that defines an entry in the matrix, in particular, an entry for row *i* and column *j* in

Now from the matrix *H*, vertex degree is defined as:

$$d_v(i) = \sum _j H(i,j)$$

This is valuable for identifying beats that are involved in many groups of morphological similarity; such groups are often associated with transitional cardiac activity.

The hyperedge degree is given by:

$$d_e(j) = \sum _i H(i,j)$$

This hypergraph technique is very effective compared to the conventional graph technique used for ECG-based arrhythmia classification problems. Firstly, hypergraphs can inherently represent high-order relationships between entities with $R: V^{(k+1)} \rightarrow \{0,1\}$ directly, making it possible to explicitly represent patterns that span multiple heartbeats. This is particularly important for issues such as ventricular bigeminy, difference in the amplitude or electrical alternans between beats, or ST segment changes over multiple consecutive beats. Graphs, on the other hand, have to represent such patterns over multiple edges, potentially making them less significant over time. Furthermore, clustering with hyperedges adequately combines similarly shaped features over heartbeats, making possible the community-based rather than isolate heartbeat examination that is common with conventional graph techniques. This facilitates more robust discrimination of subtle waveform variations that are critical for accurate arrhythmia characterization. Finally, the transitive relationship carried out through the hyperedge makes it possible to pass messages among all the connected beats. This indicates that heartbeats with some uncertainty can rely on the collective support of similar heartbeats within the same hyperedge to improve the-confidence of the classification.Fourth, “minority classes in arrhythmias, which are often underrepresented in a clinical dataset, benefit substantially from the link structure provided by hypergraphs. Rare instances of abnormal beats can aggregate knowledge about patterns that have a similar morphology across a given dataset.

6 $$\begin{aligned} \mathscr {I}(\text {rare}_i) = \sum _{j \in \mathscr {N}_k(i)} w_{ij}\,\mathscr {I}(\text {neighbor}_j) \end{aligned}$$

This amplifying the learning signal despite limited training samples and mitigating class imbalance effects that challenge conventional approaches.Finally, despite modeling complex higher-order relationships, the sparse structure of the incidence matrix $\textbf{H}$ with density $\rho = \frac{k+1}{n}$ (where *k = 5* and *n* is the number of samples) ensures computational efficiency comparable to standard graph neural networks. So, this method remains scalable for very large databases of ECGs but still takes advantage of the representational power of hypergraph methods. Instead of using graph convolutions like normal graphs, the hypergraph convolution approaches the problem by including high-order connections in the form of multiple graph paths or directions. This method enables the aggregation of information from more than two morphologically similar ECG signals that share hyperedges simultaneously^28^. Instead of relying on edges like normal graph convolutions, the hypergraph convolution employs the use of the incident matrix to transmit information regarding the hyperedges. We explain the hypergraph convolution using two degree matrices that reflect the fundamental properties of the hypergraph structure^29^. We define vertex degree matrix $D_v \in \mathbb {R}^{n \times n}$ as a diagonal matrix, with each diagonal entry indicating the number of hyperedges that a vertex (ECG beat) is involved with:

$$D_v(i,i) = \sum _j H(i,j),$$

where *n* is the number of vertices/ECG beats, *m* is the number of hyperedges, and *H* represents the incidence matrix. High-degree vertices correspond to beats with complex or ambiguous morphologies that resemble multiple arrhythmia patterns (e.g., premature atrial contractions with aberrant conduction), whereas low-degree vertices correspond to highly class-specific morphologies. The diagonal hyperedge degree matrix $\textbf{D}_e \in \mathbb {R}^{m \times m}$ measures the size (cardinality) of each hyperedge:

7 $$\begin{aligned} \textbf{D}_e(j,j) = \sum _{i=1}^{n} \textbf{H}(i,j). \end{aligned}$$

In our star-centered *k*-NN hypergraph construction with *k=5*, every hyperedge contains exactly *k+1=6* vertices; hence, all hyperedges have identical degrees. Normalization by $\textbf{D}_v^{-1}$ and $\textbf{D}_e^{-1}$ balances the aggregation, preventing highly connected beats from dominating the representation and ensuring proportional contributions from hyperedges of different sizes.

## Hypergraph convolution

The hypergraph convolution at layer *l* is defined as:

8 $$\begin{aligned} \textbf{x}^{(l+1)} = \sigma \!\left( \textbf{D}_v^{-1} \textbf{H}\, \textbf{D}_e^{-1} \textbf{H}^{\top } \textbf{x}^{(l)} \textbf{W}^{(l)} \right) , \end{aligned}$$

where $\textbf{x}^{(l)} \in \mathbb {R}^{n \times d_l}$ is the vertex feature matrix, $\textbf{W}^{(l)} \in \mathbb {R}^{d_l \times d_{l+1}}$ is a learnable weight matrix, $\sigma (\cdot )$ is the nonlinearity (LeakyReLU), and $\textbf{H}^{\top }$ is the transpose of the incidence matrix.

This operation admits a three-stage interpretation:

**1) Feature transformation:**

9 $$\begin{aligned} \textbf{z}^{(l)} = \textbf{x}^{(l)} \textbf{W}^{(l)} . \end{aligned}$$

**2) Vertex-to-hyperedge aggregation:**

10 $$\begin{aligned} \textbf{y}^{(l)} = \textbf{D}_e^{-1}\textbf{H}^{\top }\textbf{z}^{(l)}, \end{aligned}$$

with element-wise expression

11 $$\begin{aligned} y^{(l)}_{j} = \frac{1}{d_e(j)} \sum _{i : v_i \in e_j} z_i^{(l)}. \end{aligned}$$

**3) Hyperedge-to-vertex propagation:**

12 $$\begin{aligned} \textbf{x}^{(l+1)} = \sigma \!\left( \textbf{D}_v^{-1} \textbf{H}\, \textbf{y}^{(l)} \right) , \end{aligned}$$

with the vertex-wise form:

13 $$\begin{aligned} x^{(l+1)}_i = \sigma \!\left( \frac{1}{d_v(i)} \sum _{j : v_i \in e_j} y_j^{(l)} \right) . \end{aligned}$$

A standard 1D convolution computes local weighted sums over a fixed and pre-defined receptive field using a kernel^30^. In contrast, hypergraph convolution utilizes a two-stage message passing mechanism between vertices and hyperedges, followed by hyperedges and vertices^20^. In this framework, each beat updates its feature representation based on information aggregated from its *k* most morphologically similar beats across the entire dataset. This communication with the ability to go between the and hyperedges enables each heartbeat signal to receive plenty of information regarding shapes from a variety of similarity patterns. Specifically, $\Theta = D_v^{-1} H D_e^{-1} H^{\top }$ ensures that all morphologically similar heartbeats converge to consistent descriptions while smooth features are propagated with the assistance of this normalized hypergraph Laplacian. From a spectral perspective, this operator admits an intuitive interpretation, wherein low-frequency components encode global cardiac rhythms, whereas high-frequency components capture patient-specific and beat-level variations. To further stabilize training and promote effective gradient flow in deep hypergraph architectures, residual connections are incorporated into the hypergraph convolutional layers^31^.

14 $$\begin{aligned} \textbf{x}^{(l+1)} = \sigma \!\left( \textbf{D}_v^{-1} \textbf{H} \textbf{D}_e^{-1} \textbf{H}^{\top } \textbf{x}^{(l)} \textbf{W}^{(l)} \right) + \textbf{x}^{(l)}. \end{aligned}$$

**Fig. 4:**
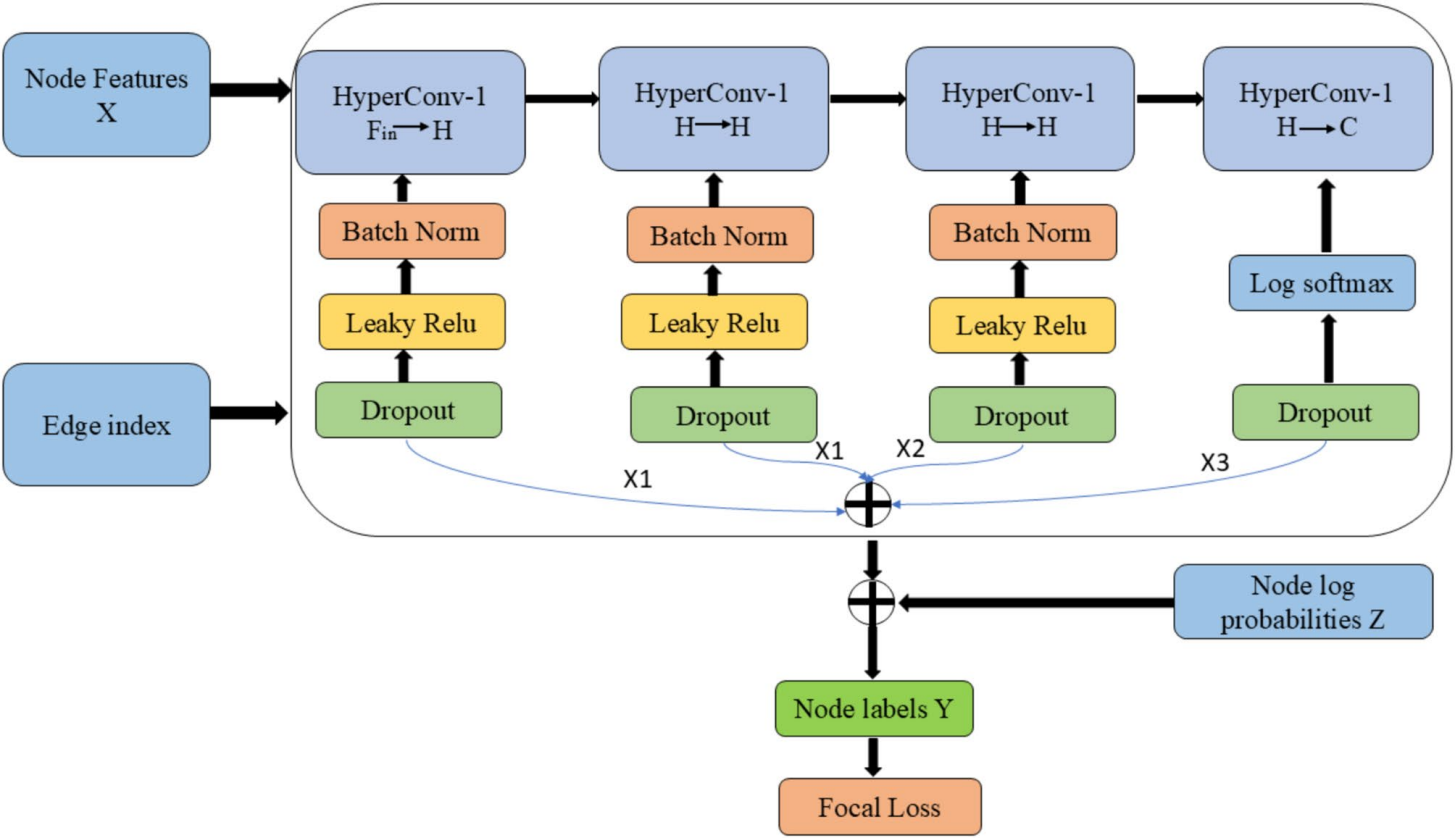
The architecture of the proposed EHGNN.

The residual mapping facilitates optimization and preserves clinically important features. The gradient with respect to layer *l* satisfies ensuring non-zero gradients even when the Jacobian becomes small and its shown in Fig. 4.

15 $$\begin{aligned} \frac{\partial \mathscr {L}}{\partial \textbf{x}^{(l)}} = \frac{\partial \mathscr {L}}{\partial \textbf{x}^{(l+1)}} \left( \frac{\partial \textbf{x}^{(l+1)}}{\partial \textbf{x}^{(l)}} + \textbf{I} \right) , \end{aligned}$$

## Training objective and loss function

The training objective of the Enhanced Hypergraph Neural Network (E-HGNN) is formulated as a multi-class classification problem designed to address the severe class imbalance present in clinical ECG arrhythmia datasets. Given a hypergraph representation $\mathscr {H} = (\mathscr {V}, \mathscr {E}, w)$ and hierarchical features learned from the CNN and hypergraph layers, the goal is to learn a mapping $f_{\Theta }: \mathbb {R}^{n \times d} \rightarrow \mathbb {R}^{n \times C}$, where *C* denotes the number of arrhythmia classes (5 for MIT-BIH AAMI, 3 for INCART). The complete training objective integrates focal loss to mitigate class imbalance and $L_2$ regularization to prevent overfitting: $\Theta ^* = \arg \min _{\Theta } \left[ \mathscr {L}_{\text {focal}}(\hat{\textbf{y}}, \textbf{y}) + \lambda \mathscr {R}(\Theta ) \right] ,$ where $\Theta = \{\theta _{\text {CNN}}, \textbf{W}^{(1)}, \textbf{W}^{(2)}, \textbf{W}^{(3)}, \textbf{W}^{(4)}, \gamma ^{(l)}, \beta ^{(l)}\}$ includes CNN parameters, hypergraph convolutional weights, and batch normalization parameters. The regularization term is given by $\mathscr {R}(\Theta ) = \sum _{\theta \in \Theta } \Vert \theta \Vert _2^2$ with $\lambda = 5 \times 10^{-4}$, while $\hat{\textbf{y}} \in \mathbb {R}^{n \times C}$ and $\textbf{y} \in \{0,1,\ldots ,C-1\}^n$ denote predicted log-probabilities and ground-truth labels, respectively.

Standard cross-entropy loss treats all samples equally, which is unsuitable for imbalanced medical datasets where normal beats vastly outnumber pathological ones. Focal loss addresses this through class-frequency weighting and difficulty-based modulation^32^. For a sample with label $y \in \{0,1,\ldots ,C-1\}$,

16 $$\begin{aligned} \mathscr {L}_{\text {focal}}^{(i)} = -\alpha _y (1 - p_y)^{\gamma } \log (p_y) \end{aligned}$$

17 $$\begin{aligned} p_y = \frac{\exp (\hat{y}_y)}{\sum _{c=0}^{C-1} \exp (\hat{y}_c)} \end{aligned}$$

Focal loss is the predicted probability of the correct class. The class weight $\alpha _y$ compensates for imbalance, and the focal loss is the focusing parameter *γ = 2.0* controls how aggressively easy examples are down-weighted. Class weights are computed via normalized inverse-frequency weighting:

18 $$\begin{aligned} \alpha _c = \frac{C \cdot N}{ n_c \sum _{j=0}^{C-1} n_j}, \end{aligned}$$

where $n_c$ is the number of samples in class *c*. The total focal loss for a batch $\mathscr {B}$ is:

19 $$\begin{aligned} \mathscr {L}_{\text {focal}} = \frac{1}{|\mathscr {B}|} \sum _{i \in \mathscr {B}} \mathscr {L}_{\text {focal}}^{(i)}. \end{aligned}$$

For optimization, we use the Adam algorithm:

20 $$\begin{aligned} m_t&= \beta _1 m_{t-1} + (1 - \beta _1) \nabla _{\Theta }\mathscr {L}, \end{aligned}$$

21 $$\begin{aligned} v_t&= \beta _2 v_{t-1} + (1 - \beta _2) \left( \nabla _{\Theta }\mathscr {L} \right) ^2, \end{aligned}$$

22 $$\begin{aligned} \hat{m}_t&= \frac{m_t}{1 - \beta _1^t}, \qquad \hat{v}_t = \frac{v_t}{1 - \beta _2^t}, \end{aligned}$$

23 $$\begin{aligned} \Theta _{t+1}&= \Theta _t - \eta \frac{\hat{m}_t}{\sqrt{\hat{v}_t} + \epsilon }, \end{aligned}$$

with hyperparameters *η = 0.01*, $\beta _1 = 0.9$, $\beta _2 = 0.999$, and $\epsilon = 10^{-8}$. To enhance convergence stability, we apply a ReduceLROnPlateau learning-rate scheduler:

$$\eta _{t+1} = {\left\{ \begin{array}{ll} 0.5\,\eta _t, & \text {if validation accuracy does not improve for 10 epochs}, \\ \eta _t, & \text {otherwise}. \end{array}\right. }$$

$L_2$ weight decay $\lambda \Vert \Theta \Vert _2^2$ encourages smoother and more generalizable parameter updates. Training and validation losses are monitored continuously, and early stopping is triggered if no validation improvement is observed for 20 epochs. The checkpoint with the highest validation accuracy is saved for final evaluation.

### Dataset and preprocessing

In this work, electrocardiographic recordings obtained from the MIT-BIH Arrhythmia Database were utilized for the systematic categorization of cardiac rhythm abnormalities^17^. This dataset is widely regarded as a representative source of diverse arrhythmias and has been extensively used as a benchmark for evaluating medical signal classification algorithms. It includes 48 ECG recordings from 47 subjects, with two recordings (201 and 202) belonging to the same individual. Each record contains two-channel ECG data that is a 30-minute record predominantly coming from lead II and lead V1. This dataset is compatible with AAMI recommendations on developing and testing beat classification-related algorithms. The five-class approach is based on the ANSI/AAMI EC57:1998/(R)2008 standard for testing and reporting performance of cardiac rhythm classification algorithms, which is the internationally recognized benchmark for single-lead ECG beat classifiers and is followed by the majority of comparative approaches in Table 2.

**Table 2:** Performance comparison of ECG arrhythmia classification methods on the MIT-BIH Arrhythmia Database.

**Reference**	**Method**	**Accuracy (%)**	**Precision (%)**	**Recall (%)**	**Specificity (%)**	**F1-Score (%)**
Hannun et al.^33^	1D CNN	94.95	95.46	94.95	—	94.97
Kachuee et al.^34^	Deep RCNN	95.90	95.20	95.10	—	—
Teijeiro et al.^35^	Clustering	97.98	91.13	—	97.66	86.26
Ghorbani Afkhami et al.^36^	GMM + Enhanced EM	96.15	88.90	—	—	—
Hammad et al.^37^	DL + GA + kNN	98.00	95.80	—	98.90	88.84
Mousavi et al.^38^	CNN + ELP	97.62	91.46	—	96.14	85.82
Xu et al.^39^	1D CNN + BiLSTM	95.90	96.34	95.90	95.92	—
Xia et al.^40^	CNN + Transformer	97.66	99.00	—	—	—
Zhao et al.^41^	ECGNN	90.00	—	—	—	82.40
Qiang et al.^14^	Conv-RGNN	88.18	77.48	76.29	—	78.73
Wang et al.^42^	IIVYA-SE-CNN-BiLSTM	95.50	95.50	95.40	—	94.90
Li et al.^43^	ResNet-18 + CBAM + Auxiliary Classifier	96.70	87.60	96.70	96.70	92.00
**Proposed Method**	**CNN + HGNN**	**98.66**	**98.64**	**98.63**	**98.64**	**98.63**

The INCART 12-lead Arrhythmia Database provided by the St. Petersburg Institute of Cardiological Technics contains 75 records that were taken from 32Holter recordings. Each record consists of 30 minutes, contains 12 standard leads, and has a sample rate of 257 Hz. The INCART recordings used lead II as their lead in our experiments. For the INCART Database, Lead II was selected among the available 12 leads to ensure anatomical consistence with the MIT-BIH Database. Five-class AAMI classification is used for the MIT-BIH Database, and three-class classification is used for the INCART Database, based on the annotation characteristics of the two datasets instead of any arbitrary modeling choice. The INCART Database has less than 15 instances of Fusion Beats and no Paced Beats at all, which is not sufficient to conduct any statistical classification. Therefore, the three clinically viable classes - Normal (N), Supraventricular Ectopic Beat (SVEB), and Ventricular Ectopic Beat (VEB) - are selected for the INCART Database, as they represent the most critical decision in any Holter-based arrhythmia screening process.

A proper ECG signal preparation is a precondition for providing proper model predictions. The ECG signal preparation includes five key steps: noise component elimination, heartbeat segmentation based on interbeat intervals, standardization of lengths of these heartbeat intervals, aggregation of heartbeat classes, and dealing with a class imbalance problem. A fourth-order Butterworth filter has been used to eliminate noise that contains lower frequency components less than 0.5 Hz, as well as noise that has higher frequency components above 50 Hz. The analog transfer function can be defined as:

24 $$\begin{aligned} H(s) = \frac{1}{\sqrt{1 + \epsilon ^2 \left( \frac{s}{j\omega _c} \right) ^{2n}}}, \end{aligned}$$

where *n = 4* is the filter order, $\omega _c$ is the cutoff frequency, and *ε* denotes the ripple factor. Zero-phase filtering was applied using forward-backward filtering to prevent phase distortion. Individual heartbeats were segmented using a physiologically informed, RR-interval-based approach (Algorithm 2)^44,45^. This approach captures complete cardiac cycles, including the P-wave, QRS complex, T-wave, and the initial portion of the subsequent P-wave.

**Fig. 5:**
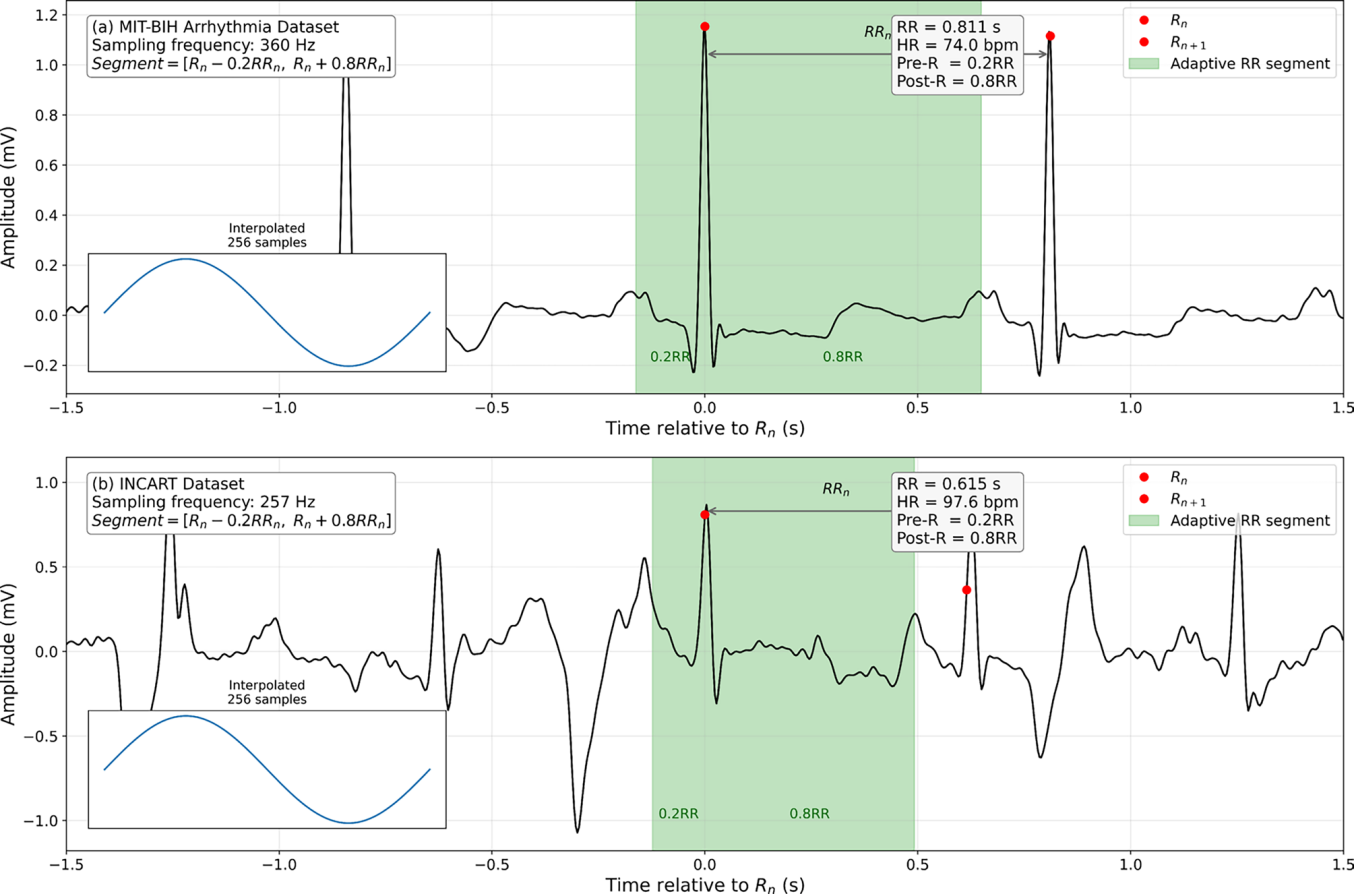
RR-adaptive ECG beat segmentation for MIT-BIH and INCART datasets.

In Fig. 5, we illustrate the preprocessing and segmentation of the MIT-BIH Arrhythmia Database. In particular, we have segments of varying lengths given by the expression $[R_n - 0.2\,RR_n, R_n + 0.8\,RR_n]$, which had to be resampled to a fixed length for model input. No form of signal-level up- or downsampling was applied to either of the considered datasets; i.e., both the MIT-BIH (360 Hz) and INCART (257 Hz) signals were processed at their native sampling rates throughout band-pass filtering and segmentation. The boundaries of the adaptive windows in Algorithm 2 are determined based on the native sampling frequency $f_s$ of the corresponding signal. In other words, the length of the raw segments varies according to both the instantaneous RR interval and the sampling rate of the signal. This issue is resolved in a single step at Line 8 of Algorithm 2, where all raw segments—regardless of arrhythmia type, heart rate, or sampling frequency—are linearly interpolated to a fixed length of exactly 256 samples. It can be seen from the data that indeed there is a significant imbalance among the classes (top-left panel). Analysis of the RR interval amplitudes demonstrates physiological variation from one beat to another that averages 0.750 sec per period and approximately 84.1 beats per minute. There is successful segmentation of a whole heartbeat cycle among all arrhythmias, and there is notable variation among the waveform samples.

**Algorithm 2:**
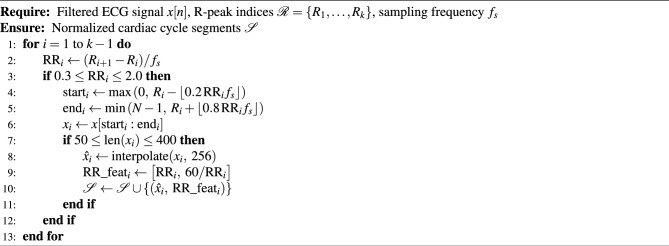
RR-interval–based cardiac cycle segmentation.

## Implementation details

This work proposes a hybrid deep learning framework which integrates CNNs to extract hierarchical features with enhanced hypergraph neural networks to capture higher-order relationships for ECG arrhythmia classification. The framework is evaluated in an end-to-end manner, encompassing signal filtering, heartbeat segmentation, hypergraph construction, model training, and performance evaluation. Experimental validation is conducted on both the MIT-BIH Arrhythmia Database and the INCART 12-lead Arrhythmia Database to demonstrate the effectiveness and generalizability of the proposed approach.

The raw ECG signals are first denoised using a fourth-order Butterworth bandpass filter. For MIT-BIH, a passband of 0.5–50 Hz is used at a sampling frequency of $f_s = 360$ Hz, whereas for INCART a passband of 0.5–40 Hz is applied at $f_s = 257$ Hz. A 50/60 Hz notch filter further suppresses powerline interference. The lower cut-off of 0.5 Hz effectively removes baseline wander due to respiration and motion, while the upper cut-off (40–50 Hz) attenuates electromyographic and instrumentation noise. Zero-phase filtering using the forward–backward approach avoids temporal distortion of clinically important PQRST morphology. ECG heartbeat segmentation is performed using annotation-anchored fixed windows. For each annotated R-peak $p_i$, a window of length *L=256* samples is extracted:

$$x_i[n] = x\!\left( \max \!\left( 0,\, p_i - \left\lfloor \frac{L}{2} \right\rfloor + n \right) \right) ,\quad n=0,1,\ldots ,L-1,$$

where *x*[*k*] is the filtered ECG signal and the *\max* function prevents out-of-bound indexing. The 256-sample window corresponds to approximately 0.711 s for MIT-BIH and 0.996 s for INCART, capturing the full PQRST complex across heart rates ranging from 40–150 bpm. For INCART 12-lead recordings, Lead II is selected to maintain consistency with MIT-BIH.

Each extracted segment is standardized using z-score normalization:

$$\tilde{x}_i[n] = \frac{x_i[n] - \mu _i}{\sigma _i + \epsilon },$$

where

$$\mu _i = \frac{1}{L}\sum _{n=0}^{L-1} x_i[n],\qquad \sigma _i = \sqrt{\frac{1}{L}\sum _{n=0}^{L-1}(x_i[n]-\mu _i)^2},$$

and $\epsilon = 10^{-8}$. This normalization removes inter-patient and inter-session amplitude variations, allowing the CNN to focus on waveform morphology. Annotations are mapped to AAMI classes, where the class distribution is highly imbalanced (Normal $\approx 75\%$, Fusion *<1%*). To mitigate imbalance in MIT-BIH, a conservative SMOTE strategy is applied:

$$\hat{n}_c = \min \!\left( 1.5\,n_c,\, n_c + 1000\right) - n_c,$$

where $n_c$ is the original class count and $\hat{n}_c$ is the number of synthetic samples generated. Synthetic samples are produced via linear interpolation:

$$\tilde{x} = x_i + \lambda (x_j - x_i),\qquad \lambda \sim \mathscr {U}(0,1),$$

where $x_j$ is one of the $k=\min (5,n_c -1)$ nearest neighbors of $x_i$. For INCART, physiologically valid augmentations are used instead of SMOTE, including amplitude scaling, Gaussian noise injection, temporal warping (resampling factor $\beta \sim \mathscr {U}(0.94,1.06)$), and baseline modulation using sinusoidal components with frequencies $f\sim \mathscr {U}(0.1,0.5)$ Hz^46,47^.

The CNN feature extractor consists of four sequential 1D convolutional blocks with filter counts 64, 128, 256, and 512, and kernel sizes 7, 5, 3, and 3, respectively. Each layer is followed by batch normalization (momentum = 0.9, $\epsilon =10^{-5}$), ReLU activation, and dropout (*p=0.2*). Hierarchical average pooling (pool size 4, stride 2) is applied twice, reducing the temporal dimension from 256 to approximately 46 samples. Features are flattened and passed through a dense layer with 768 neurons (ReLU, dropout *p=0.3*). Two additional RR interval features (interval duration and instantaneous heart rate) are appended, yielding final 770-dimensional feature vectors. Hypergraphs are constructed using a 5-nearest-neighbor scheme based on cosine similarity in the 770-dimensional feature space. The EHGNN comprises four hypergraph convolution layers with hidden dimension 128, residual connections from layers 2–4, batch normalization (momentum = 0.9), LeakyReLU activation (slope = 0.2), and dropout (*p=0.3*). The final layer produces log-probabilities over the arrhythmia classes via log-softmax, with *C=5* for MIT-BIH and *C=3* for INCART^48^.

Model optimization is performed using the Adam optimizer with initial learning rate $\eta _0 = 0.01$, $\beta _1 = 0.9$, $\beta _2 = 0.999$, and weight decay $\lambda = 5\times 10^{-4}$. A ReduceLROnPlateau scheduler halves the learning rate when validation accuracy plateaus for 10 epochs. Focal Loss with focusing parameter *γ =2.0* and inverse-frequency class weights $\alpha _c$ is used to address imbalance. For MIT-BIH, the approximate class weights are: $\alpha _S \approx 2.5, \alpha _V \approx 1.8, \alpha _F \approx 8.5, \alpha _Q \approx 1.2.$ Training uses batch size 256 and a stratified 80/20 train–test split. The maximum epochs are 200 for MIT-BIH and 300 for INCART, with early stopping (patience = 20). Random seed 42 is used for reproducibility. The CNN is implemented in TensorFlow 2.12 (Keras), while the EHGNN is implemented in PyTorch 2.0 using PyTorch Geometric 2.3. All experiments are conducted on a Dell Precision 3660 workstation equipped with an Intel Core i7-12700 CPU (12 cores, 20 threads), 128 GB DDR4 RAM, an NVIDIA RTX A4000 GPU (16 GB), and Windows 11 Pro. Training typically converges within 200 epochs for MIT-BIH and 200 epochs for INCART. Model performance is evaluated using accuracy, precision, recall, F1-score, confusion matrices, and ROC–AUC curves, computed on the held-out 20% test set.

##  Evaluation metrics

The performance evaluation of the model is presented with a set of comprehensive metrics that collectively demonstrate the accuracy, class performance, and usefulness of the solutions. Accuracy measures the overall correct assignment of the heartbeat patterns, representing the ability of the system to assign patterns to the correct class on average. Precision measures the validity of the predicted arrhythmic events, analyzing the positive predictions to ascertain the likelihood that the actual predictions are true, illustrating the ability of the system to avoid raising false positive alerts that would affect the clinical validity of the predictions. The value of recall, which corresponds to the sensitivity, provides the ability to analyze the actual performance of the predictions to ensure the clinically significant events are reported, hence reflecting the capacity to identify the real occurrences without omitting the significant ones. The use of an F1 value, which averages the precision and recall, provides a solution that represents a complete performance evaluation without the imprecise nature introduced by the imbalanced dataset that could suggest a different performance value based on the use of accuracy alone. The use of the confusion matrices helps to evaluate the relative performance of the true class compared to the predicted class, allowing the identification of potential errors, for instance, supra ventricular beats that are classified under the normal, along with the errors that are introduced due to the overlap, which are the fusion beats classified under the ventricular ectopics, thus enabling the correction of the performance of the models. The evaluation of the performance further uses the ROC calculations for all the classes on a one-vs.-rest approach, along with the micro-avged ROC to ascertain the ability of the models to discriminate without consideration of the threshold value applied. The evaluation of the performance uses the stratified set that represents 20% of the entire dataset without any consideration to allow an unbiased performance on unseen data that was not part of the training or optimization time.

## Results

### A. Classification performance on the MIT-BIH database

**Fig. 6:**
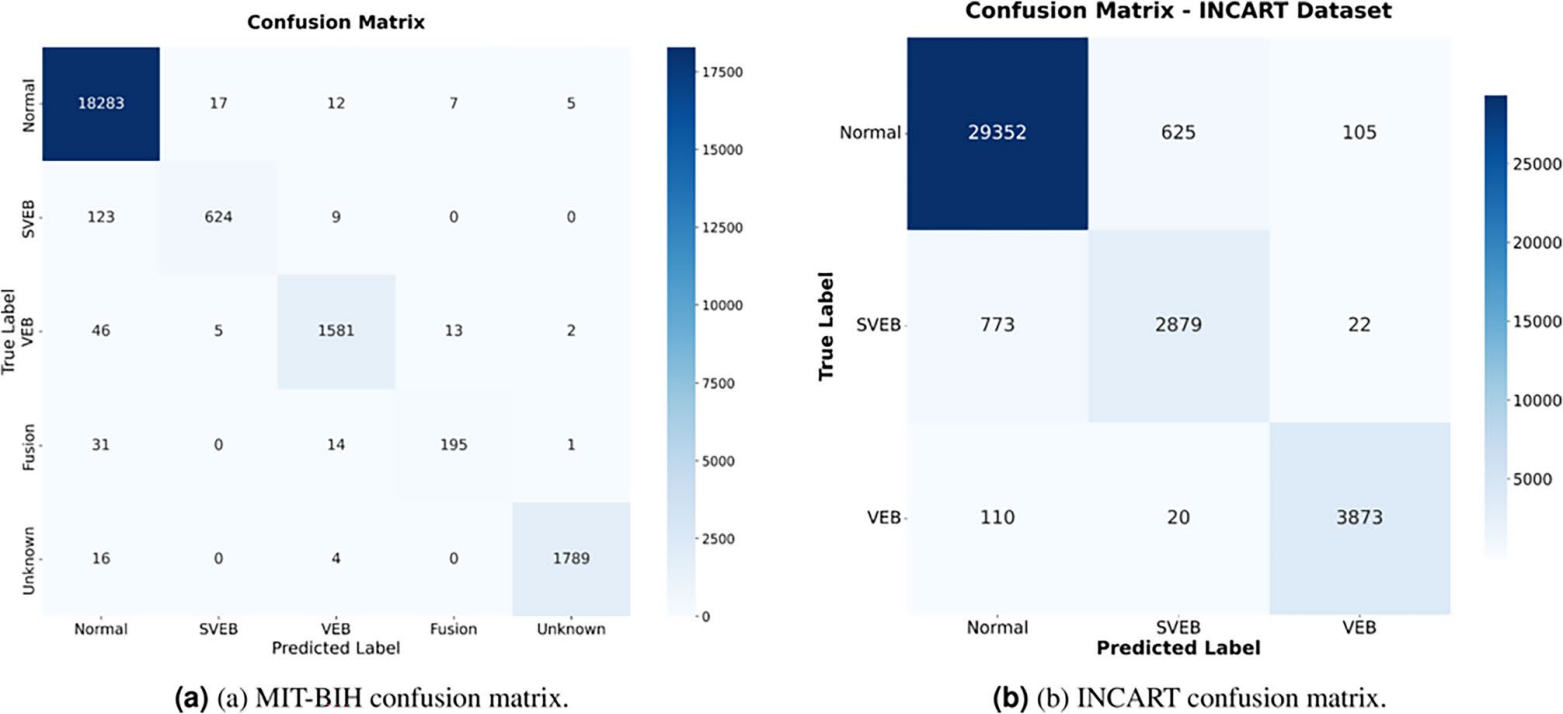
Confusion matrices for both datasets: (**a**) MIT-BIH confusion matrix showing five-class AAMI performance. (**b**) INCART confusion matrix demonstrating three-class generalization capability.

In Fig. 6(a), the confusion matrix for the MIT-BIH test set shows how well the classifier is able to distinctly classify the five classes of AAMI arrhythmias with hardly any confusion. A high diagonal value of the matrix is an indicator of well-defined class boundaries. Normal beats are similarly close to being fully correctly classified, with only 40 errors out of 18,324 as incorrect, of which the majority are misclassified into other categories. This yields a high recall of 99.78%. Ventricular ectopic beats are similarly close to being fully correct, with 1,581 correct identifications out of 1,647 samples for a recall of 95.99%. Most of these are incorrectly classified as Normal beats because of the existence of samples with small QRS complexes and a morphology closely resembling the morphology of SVEB beats. SVEB is similarly the hardest category of beats for humans to classify: only 624 out of 756 samples are correctly identified for a recall of 82.54%. Most of these misclassifications are of SVEB as Normal beats because of the existence of premature atrial contractions with abnormal conduction or Normal beats with Bundle Branch Block pattern morphologies. There are relatively few samples of beats of the Fusion category (241 samples), but these beats are still identified correctly in 195 samples for a recall of 80.91% as depicted in Table 3. Most of these misclassifications lie amongst the Normal, SVEB, VEB, or Unknown categories of beats. This is an indicator of how high-order connections of the hypergraph helped understand commonalities amongst morphologies of beats of differing types. Finally, there is an almost perfect classification of Unknown beats, with 1,789 samples being correctly identified out of 1,809 for a recall of 98.89%. There are 20 samples of this category that are identified as wrong.

**Table 3:** Classification performance on MIT-BIH Database.

**Class**	**Precision**	**Recall**	**F1-score**	**Support**
Normal	0.9883	0.9978	0.9930	18,324
SVEB	0.9659	0.8254	0.8902	756
VEB	0.9759	0.9599	0.9679	1,647
Fusion	0.9070	0.8091	0.8553	241
Unknown	0.9955	0.9889	0.9922	1,809
Accuracy	0.9866			22,777
Macro Avg	0.9665	0.9162	0.9397	22,777
Weighted Avg	0.9864	0.9866	0.9863	22,777

**Fig. 7:**
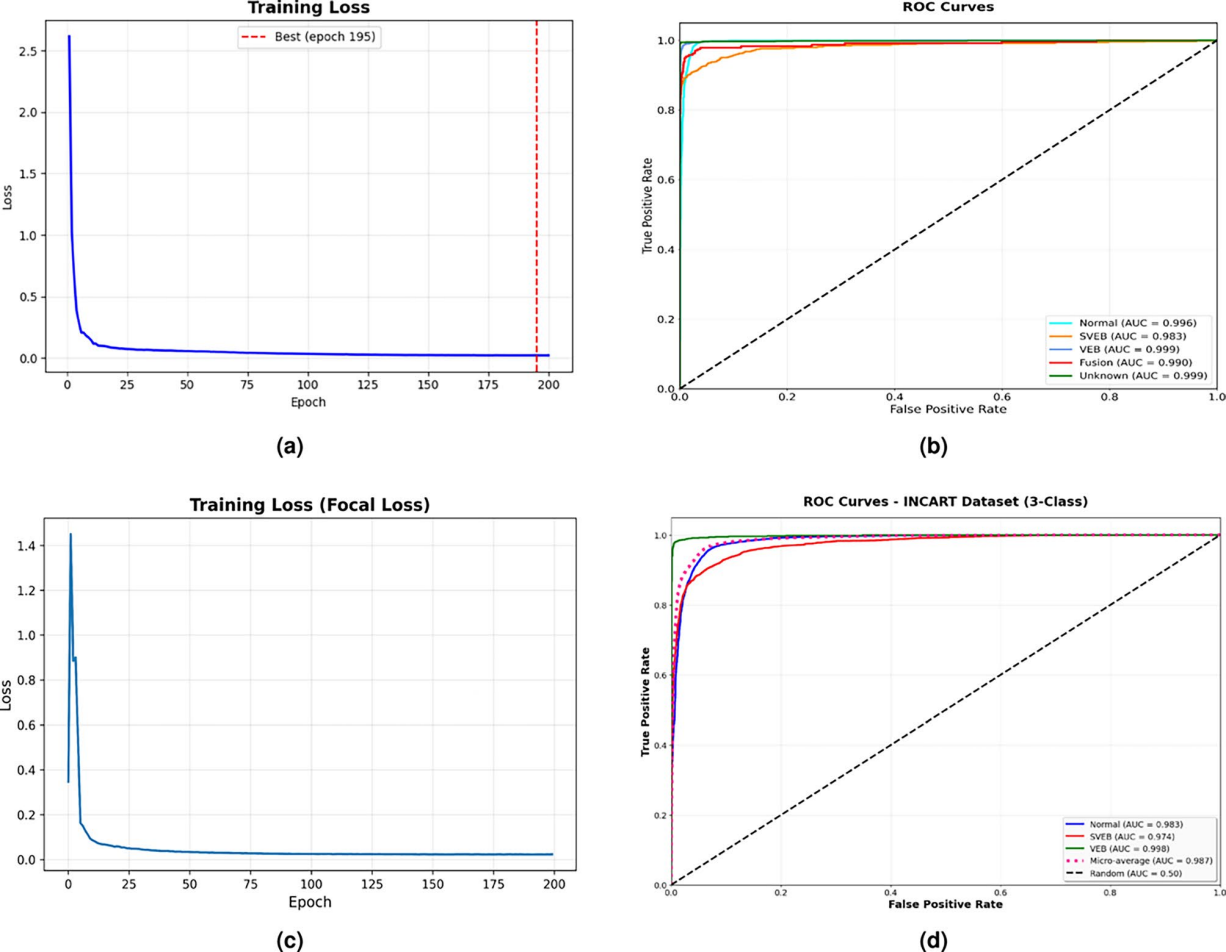
(**a**) MIT-BIH training loss convergence; (**b**) ROC curves for multi-class classification on MIT-BIH; (**c**) focal loss convergence during training; (**d**) class-wise ROC curves on the INCART dataset.

Referring to training dynamics, Fig. 7(a) depicts that the training loss declines steeply from 2.5 to 0.1 in the range of the first 50 epochs, after which it becomes relatively stable. The validation accuracy rises to approximately 95% in those first 50 epochs, after which it stabilizes to attain a maximum of 98.66% accuracy at epoch 195. This explains that the model converges to an optimal point where there is no overfitting. The stability of both training and validation accuracy further indicates that regularization techniques such as dropout, weight decay, and normalization are functioning effectively.

### B.INCART three-class classification and cross-database generalization

The proposed approach uses the EHGNN on the INCART 12-lead ECG database with three categories: Normal, SVEB, and VEB. The confusion matrix for INCART (Fig. 6(b)) portrays good results with an accuracy of 95.62% despite large differences in recording rates (257 Hz and 360 Hz), leads (12 and 2), and patients (Europe and North America). The number of correct identifications for the normal patterns is 29,351 out of 30,082, showing a sensitivity of 97.57%. The major mistakes belong to class SVEB with 683 occurrences. Then come VEB patterns with 48 mistakes. In class SVEB, 2,879 out of 3,674 patterns have been identified correctly, yielding a sensitivity of 78.36%. Most mistakes belong to class Normal with 730 occurrences. These patterns were identified due to strong morphological links. VEB patterns have been identified aptly with 3,870 out of 4,003 patterns tested; its sensitivity is 96.75%, showing good adaptation to the traits of INCART’s features.

Convergence of training on the INCART dataset (Fig. 7(c)) also reveals fast acceleration, where the training loss significantly declines in the first 25 epochs. The optimal operating point (epoch 106, red dotted line) represents a validation accuracy of approximately 95.62%. The slight reduction in performance between the INCART and MIT-BIH datasets (95.62% vs. 98.66%) stems from cross-database evaluation issues, such as reduced temporal resolution, couplings among the 12 channels, as well as differences in ECG morphology between the target population groups. However, it should be noted that our method succeeds in sustaining accuracy values above 95% on both datasets, revealing effective detection of minority classes on both datasets. These observations are validated through the classification accuracy reported in Table 4.

**Table 4:** Classification report of INCART dataset.

Class	Precision	Recall	F1-Score	Support
Normal	0.9708	0.9757	0.9733	30082
SVEB	0.8170	0.7836	0.7999	3674
VEB	0.9683	0.9675	0.9679	4003
**Accuracy**	—	—	0.9562	37759
Macro Avg	0.9187	0.9090	0.9137	37759
Weighted Avg	0.9556	0.9562	0.9558	37759

### C. Ablation study

The ablation study in Table 5 is designed to measure the impact of each module of the proposed model. By replacing the hypergraph-based EHGNN with a regular GNN, the recall is reduced significantly to 81.03% and the F1-score is reduced to 86.06%, which validates the importance of the proposed hypergraph-based higher-order relational modelling for capturing the dependencies between ECG beats. By removing the SMOTE oversampling technique, the recall is reduced to 97.25% and the accuracy is reduced to 96.17%, thereby validating the importance of the proposed oversampling technique for discriminative modelling of minority arrhythmias. By removing the residual connections, the accuracy is reduced to 97.22% and the F1-score is reduced to 96.63%, while the mean gradient norm is reduced from 0.3118 to 0.1627 and the minimum gradient norm is reduced from 0.0166 to 0.0156, thereby validating the importance of the proposed residual connections in maintaining gradient flow and preventing vanishing gradients in deeper networks as per Equation 18. The proposed model achieves the highest accuracy of 98.66% and the highest F1-score of 98.63% across all metrics, thereby validating the importance of each proposed module in improving the overall classification performance.

**Table 5:** Ablation study of the proposed model.

Model	Precision (%)	Recall (%)	F1-Score (%)	Accuracy (%)
GNN + CNN	94.41	81.03	86.06	94.43
Proposed (without SMOTE)	95.51	97.25	97.22	96.17
Proposed (without Residual)	97.42	96.62	96.63	97.22
Proposed	**98.64**	**98.63**	**98.63**	**98.66**

## Comparative analysis

For the purpose of analyzing our EHGNN approach, we compare our proposal directly with the top ten arrhythmia classification models on the ECG signals using the MIT-BIH Arrhythmia Database. As evident from the comparison table provided in Table 2, our hybrid approach using CNN and HGNN achieves an accuracy of 98.66%, outclassing the previous best result of 97.96% provided by Hammad et al.^37^ by 0.68 percentage points, using the same MIT-BIH Arrhythmia Database with the application of a deep learning approach using an optimization technique fueled by the genetic algorithm and k-NN. It is also able to achieve precision, recall, and F1-score values of 98.64%, 98.66%, and 98.63%, constituting the best-ever recorded performance in the literature for the respective parameters. Early deep learning methods solely based on CNNs, such as Hannun et al.^33^ with 94.95% accuracy using 1D CNN and Kachuee et al.^34^ with 95.9% accuracy using Deep RCNN, demonstrated the feasibility of end-to-end learning for ECG classification but had limited capability in capturing temporal dependencies and handling class imbalance, resulting in F1-scores around 95%. Hybrid spatial-temporal architectures that combined CNNs with recurrent components, such as Xu et al.^39^ with 95.90% accuracy and 96.34% precision using 1D CNN+BiLSTM, improved temporal modeling but remained vulnerable to vanishing gradient problems and showed moderate performance on minority classes.

**Fig. 8:**
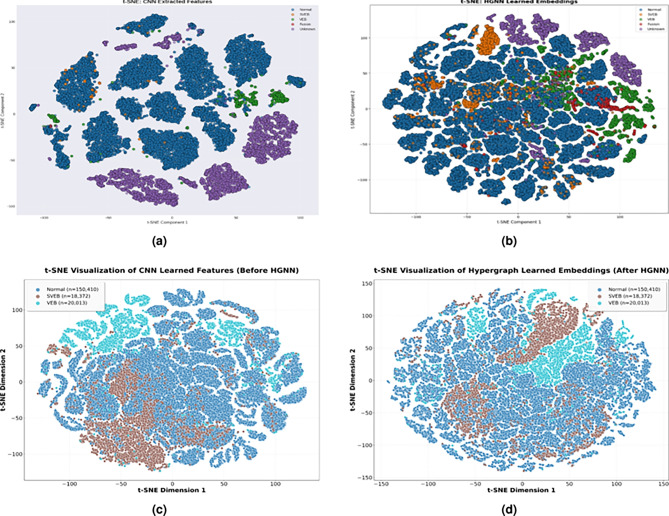
t-SNE visualization of INCART dataset features. (**a**,**c**) CNN features before HGNN processing. (**b**,**d**) HGNN embeddings showing improved class separability. Classes: Normal (blue), SVEB (orange), VEB (green).

More modern approaches based on increasingly complex architectural units provide only marginal performance improvements. The work of Xia *et al.* achieved an accuracy of 97.66% with an unusually high precision of 99.00% using a CNN–Transformer architecture, indicating strong false-positive suppression. However, recall and F1-score were not reported, suggesting potential compromises in sensitivity, particularly for minority-class arrhythmias. Traditional methods relying heavily on feature engineering, such as Teijeiro et al.^35^ and Ghorbani Afkhami et al.^36^, show respectable but limited performance. Graph neural network approaches—although motivated by the need to model inter-beat relationships—exhibit significant performance gaps: Zhao et al.’s ECGNN^41^ achieves only 90.0% accuracy with 82.4% F1-score, and Qiang et al.’s Conv-RGNN^14^ reports 88.18% accuracy with a 78.73% F1-score. These results indicate that traditional pairwise graph structures fail to capture the complex higher-order dependencies characteristic of ECG dynamics. The substantially higher performance of our hypergraph-based method (98.66% accuracy, 98.63% F1-score) over conventional graph approaches (88–90% accuracy, 78–82% F1-score) strongly supports our core hypothesis: hypergraph convolutions effectively model higher-order relationships among multiple morphologically similar heartbeats, capturing clinically significant multi-beat patterns (e.g., bigeminy, electrical alternans, progressive morphological changes) that cannot be represented by standard pairwise graph edges. The t-SNE projections shown in Fig. 8 describe differences in feature representations before (a, c) and after hypergraph neural network processing (b, d) in the INCART dataset^49,50^. The features derived via CNN (left) demonstrate partial clustering with highly overlapping classes for Normal (blue), SVEB (orange), and VEB (green) classes in particular. Clustering after HGNN processing (right) clearly indicates improved feature separation, with each class well encapsulated in compact and clearly distinguishable clusters. This provides a visual indication of how the HGNN enhances feature granularity by integrating local temporal cues with global relational awareness through hypergraph modeling, enabling the identification of relationships that conventional CNN-based features alone are unable to capture. Thus, it can be stated that a well-balanced performance is achieved through the approach as it attains a precision of 98.66% to prevent False Positives, a recall of 98.63% to prevent False Negatives, and an F1 score of 98.63% for precision and recall trade-off. In clinical applications, both false positives and false negatives are equally critical in terms of patient-level clinical severity. The framework is designed by constructing CNN-based hierarchical feature representations, validated through cosine similarity-based hypergraph construction, enhanced by improving residual hypergraph convolutional layers (R-HGConvs) using residual connections, and further strengthened by employing focal loss to address class imbalance. Through extensive empirical validation on the MIT-BIH dataset, the proposed EHGNN effectively addresses the key limitations of existing methods, including CNNs, RNNs, transformers, and conventional GNNs, for ECG-based arrhythmia classification.

## Discussion

The Enhanced Hypergraph Neural Network (EHGNN) is very effective in classification tasks for both the MIT-BIH and INCART collections with an accuracy of 98.68% and 95.46%, respectively, and overall performance is excellent for all classes too. Especially worthy of note is that all the more challenging classes for classification are handled very effectively too: Ventricular Ectopic Beats (VEB) with 96.79% F1-Score, Fusion Beats with 85.53% F1-Score, and SupraVentricular Ectopic Beats (SVEB) with 89.02% F1-Score. The key insight of this approach is its effective combination of convolutional neural networks for morphological feature learning at multiple scales with Hypergraph Neural Networks capable of capturing complex inter-beat interactions. The use of hyperedges which simultaneously connect five morphologically similar samples helps to effectively capture clinically significant temporal patterns like Ventricular Bigeminy, Electrical Alternans, or the evolution of ST-segment activity which very intrinsically require context about multiple instances of beats in time. The use of Cosine Similarity for Hypergraph construction provides an additional benefit of being invariant to scales to help remove the effects of variability due to different scales or patient physiology for the same morphological feature being matched—irrespective of the actual amplitude.

Additionally, by using focal loss with class-specific weights and difficulty adjustment, class imbalance can also be mitigated to some extent because it gives less priority to simply classified and abundant instances of some classes and concentrates more on those of other classes that are more difficult to classify. Residual learning blocks, BN layers, Leaky ReLU activation functions, and judicious use of dropout layers are also quite important in making training stable for the proposed deeper hypergraph structure without causing instabilities observed in their predecessors in the ECG graphs domain. The slightly lower recall on the fusion beat series is because such beats involve the integration of ventricular and supraventricular activation, resulting in intermittent waveforms that vary highly on the morphological spectrum. These waveforms do not belong to tight groups. Moreover, the data is sparse (only 241 instances, or 1.06%), making them difficult to identify, even for the expertise of a cardiologist. Although the databases vary on sampling rates, configurations, patients, and level of detail on labeling, the robust performance on the INCART databases demonstrates well the ability to generalize.

A number of directions exist in terms of further work based on the current limitations. Firstly, the utilization of only a single-lead modality falls short of the spatial information conveyed within the multi-lead ECGs. Secondly, to fully explore patient- or rhythm-dependent associations, it may be desirable to maintain the fixed size of the chosen neighbors within the hypergraph, *k=5*. Thirdly, from a real-world application perspective, offline analysis carries myriad limitations. Fourthly, interpretability lags rule-based methods. Lastly, it proves necessary to extend validation to different sources of databases to include varied demographics, types of recordings, before actual clinical applications. One may consider adaptive hypergraph apparatuses that could automatically change the value of the fixed size of the neighbors within the hypergraph. Additionally, online machine learning strategies to suit real-time processing of ECG signals could further these studies. Furthermore, attribution methods based on hypergraphs to discuss the morphological components underlying the decisions could significantly improve these studies.

**Fig. 9:**
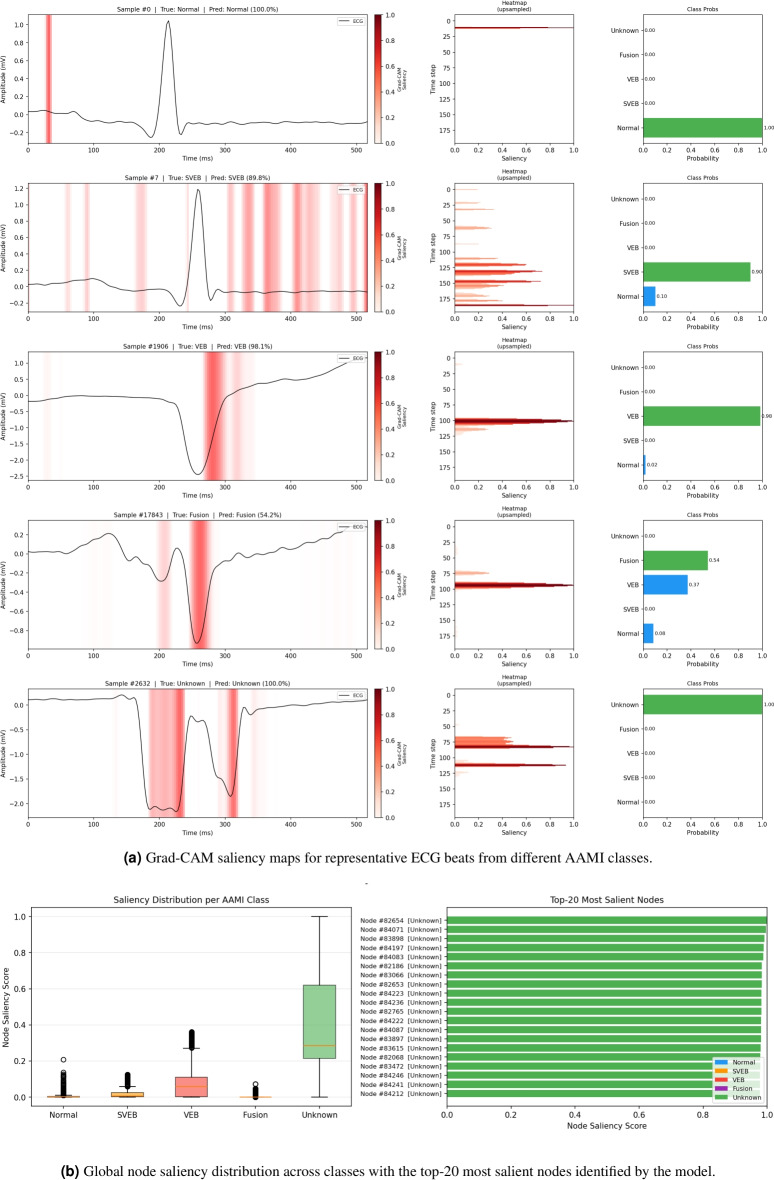
Grad-CAM saliency maps for representative beats per AAMI class (**a**) and global node saliency distribution across classes with top-20 most salient nodes (**b**).

To further illustrate the decision-making behaviour of the proposed model, the Grad-CAM saliency maps of the EHGNN model for one exemplary beat per AAMI class are depicted in Fig. 9a. It can be observed that the saliency maps of the EHGNN model show significant attention focused on the clinically relevant temporal regions of the ECG signal. Specifically, Normal-class beats show high saliency values in the QRS complex, VEB-class beats show high saliency values in the wide ventricular deflection area, Fusion-class beats show high saliency values across the entire QRS and ST segments of the signal, and Unknown-class (Paced) beats show high saliency values in the pacing spike region. Moreover, the global node saliency distribution of the hypergraph, depicted in Fig. 9b, shows that the Unknown class exhibits the highest saliency values and that the top 20 most salient nodes in the hypergraph belong exclusively to the Unknown class. This observation indicates that the EHGNN model assigns the highest relational importance to beats that deviate most significantly from the dominant Normal-class morphology.

## Clinical relevence

The proposed EHGNN model is not only focused on achieving a good accuracy rate but is valuable to the clinical community due to its ability to identify potentially life-threatening arrhythmias that are both rare and hazardous to the patient’s health. It performed exceptionally on the detection of the minority class of ventricular ectopic and fusion beats (with 96.79% and 85.53% F1-scores, respectively). These are relatively rare and highly variable signals that are, nevertheless, of great clinical significance. Ventricular ectopy may indicate structural heart disease, electrolyte imbalances, and/or drug toxicity. However, among patients with a reduced EF and those who have had a heart attack, a high incidence of ventricular ectopy confers a substantially increased risk of sudden cardiac death. Therefore, a highly sensitive tool to identify such dangers is of great use. It should be remembered that fusion beats are also of equal importance, reflecting co-existing pacing and the potential diagnoses of AV dissociation, complete heart block, and/or ventricular tachycardia. In such situations, timely detection is vital. An EHGNN model that is both highly sensitive and has a good positive predictiveness prevents potentially lethal omission detections.

Besides the distinct classes identified, the performance of the model for the different kinds of arrhythmic events collectively is balanced. The macro-averaged precision, recall, and F1-score for the model is 96.65%, 91.62%, and 93.97%, respectively. These scores are practically equal to the weighted averages of 98.64%, 98.66%, and 98.63%, which implies that the technique is universally effective, irrespective of the prevalence rates of the various kinds of events. This makes the technique useful for almost any application, be it automatic ECG recording for ambulatory patients and ICU patients or risk evaluation and EHR assistance. Another validation task conducted on a different database, named INCART, emphasizes its translational ability. It also reached 95.46% accuracy levels despite significant domain shifts with regard to sampling rates (360 Hz vs. 257 Hz), lead configurations (2-lead ambulatory vs. 12-lead ECGs), as well as with subjects’ geographical confinements (North America vs. Europe).In typical ECG applications, validating significant domain shifts with regard to ECG leads is particularly challenging for most algorithms. Nevertheless, EHGNN performs effectively for MIT-BIH as well. It is ready for real-time surveillance, wearables, and population screening. Ultimately, the generalization capability and diagnostic equity of EHGNN support its potential to democratize access to expert-level ECG interpretation globally, reduce disparities in cardiovascular disease detection, and enhance patient safety across diverse clinical and resource settings.

## Conclusion

This work proposes a novel combination of a deep learning configuration that pairs CNNs for the morphological characterization of heartbeats with an Enhanced Hypergraph Neural Network (EHGNN) for encoding strong relations between the beats. The proposed deep configuration has achieved state-of-the-art performance on ECG arrhythmia classification tasks, with 98.66% accuracy and an F1-score of 98.63% on the MIT-BIH Arrhythmia Database, and a 95.62% accuracy level on the INCART Database. The essence of the innovation is the hypergraph concept itself, as it connects different morphologically similar heartbeats via hyperedges, reflecting the complex dependencies between rhythms, which cannot be represented by the simple connections used in traditional graphs. It combines a number of different elements in order to address the long-standing problem of ECG classification, including CNN-based encoders that extract multi-scale features from individual beats, a hypergraph constructed based on the similarity of morphologies between scales using the cosine similarity function allowing for scale-invariant matching, residual hypergraph convolutions to enhance the flow of information at a deeper level and to couple signals from neighboring regions, residual blocks, and focal loss with class-aware weights for dealing with significant class imbalance, emphasizing the difficult rhythms for classification.

## Data Availability

The experimental data supporting the results and findings of this study are publicly available from the PhysioNet repository. The MIT-BIH Arrhythmia Database can be accessed at https://physionet.org/content/mitdb/1.0.0/, while the INCART 12-lead Arrhythmia Database is available at https://physionet.org/content/incartdb/1.0.0/.

## References

[1] World Health Organization. Cardiovascular diseases (CVDs). (2022). https://www.who.int/news-room/fact-sheets/detail/cardiovascular-diseases-(cvds)

[2] Bayés de Luna, A. *Basic Electrocardiography* (Wiley, 2007). 10.1002/9780470692622.

[3] Irungu, J., Oladunni, T., Denis, M., Ososanya, E. & Muriithi, R. A CNN transfer learning electrocardiogram (ECG) signal approach to predict COVID-19. *Proc. 15th Int. Conf. Comput. Autom. Eng.* 367–371 (2023). 10.1109/ICCAE56788.2023.10111114

[4] Tang, X., Ma, Z., Hu, Q. & Tang, W. A real-time arrhythmia heartbeats classification algorithm. *IEEE Trans. Biomed. Eng.* **67**, 978–986. 10.1109/TBME.2019.2926104 (2020). 31265382 10.1109/TBME.2019.2926104

[5] Martis, R. J., Acharya, U. R., Mandana, K., Ray, A. K. & Chakraborty, C. Cardiac decision making using higher order spectra. *Biomed. Signal Process. Control* **8**, 193–203 (2013).

[6] Coutinho, D. P. et al. Novel fiducial and non-fiducial approaches to ECG-based biometric systems. *IET Biom.* **2**, 64–75 (2013).

[7] Goovaerts, G. et al. A machine-learning approach for detection and quantification of QRS fragmentation. *IEEE J. Biomed. Health Inform.* **23**, 1980–1989 (2019). 30371397 10.1109/JBHI.2018.2878492

[8] Jiang, Y. et al. A comprehensive survey of deep learning based ECG classification. *IEEE Access* **9**, 65017–65033 (2021).

[9] Kiranyaz, S., Ince, T. & Gabbouj, M. Real-time patient-specific ECG classification using 1D CNNs. *IEEE Trans. Biomed. Eng.* **63**, 664–675. 10.1109/TBME.2015.2468589 (2016). 26285054 10.1109/TBME.2015.2468589

[10] Zhang, Y., Li, P., Cheng, L., Li, M. & Li, H. Attention-based multiscale spatial–temporal CNN for EEG decoding. *IEEE Trans. Consum. Electron.* **70**, 2423–2434. 10.1109/TCE.2023.3330423 (2024).

[11] Butt, F. S., Wagner, M. F., Schäfer, J. & Ullate, D. G. Toward automated feature extraction for deep learning classification of ECG signals. *IEEE Access* **10**, 118601–118616 (2022).

[12] Yan, G., Liang, S., Zhang, Y. & Liu, F. Fusing transformer models with temporal features for ECG classification. *Proc. IEEE Int. Conf. Bioinformatics Biomed.* 898–905 (2019). 10.1109/BIBM47256.2019.8983326

[13] Anwar, A., Khalifa, Y., Coyle, J. L. & Sejdic, E. Transformers in biosignal analysis: A review. *Inf. Fusion* **114**, 102697. 10.1016/j.inffus.2024.102697 (2025).

[14] Qiang, Y. et al. Conv-RGNN: An efficient convolutional residual GNN for ECG classification. *Comput. Methods Programs Biomed.* **257**, 108406 (2024). 39241329 10.1016/j.cmpb.2024.108406

[15] Shi, H. et al. Hypergraph-induced convolutional networks for visual classification. *IEEE Trans. Neural Netw. Learn. Syst.* **30**, 2963–2972 (2019). 30295630 10.1109/TNNLS.2018.2869747

[16] Yuan, S. et al. Hypergraph and cross-attention-based unsupervised domain adaptation framework for cross-domain myocardial infarction localization. *Inf. Sci.* **633**, 245–263. 10.1016/j.ins.2023.03.078 (2023).

[17] Moody, G. B. & Mark, R. G. The impact of the MIT-BIH Arrhythmia Database. *IEEE Eng. Med. Biol. Mag.* **20**, 45–50. 10.1109/51.932724 (2001). 11446209 10.1109/51.932724

[18] Goldberger, A. L. et al. PhysioBank, PhysioToolkit, and PhysioNet: Components of a new research resource for complex physiologic signals. *Circulation* **101**(23), e215–e220. 10.1161/01.cir.101.23.e215 (2000). 10851218 10.1161/01.cir.101.23.e215

[19] Dong, Y., Liu, Q., Du, B. & Zhang, L. Weighted feature fusion of CNN and GAT for hyperspectral image classification. *IEEE Trans. Image Process.* **31**, 1559–1572 (2022). 35077363 10.1109/TIP.2022.3144017

[20] Feng, Y., You, H., Zhang, Z., Ji, R. & Gao, Y. *Hypergraph neural networks. AAAI* **33**, 3558–3565 (2019).

[21] Aggarwal, C. C., Hinneburg, A. & Keim, D. A. On the surprising behavior of distance metrics in high dimensional space. In *Proc. Int. Conf. Database Theory (ICDT)*, Lecture Notes in Computer Science, vol. 1973, pp. 420–434, 10.1007/3-540-44503-X_27. (Berlin, Heidelberg: Springer, 2001).

[22] Luo, C. et al. Cosine normalization: Using cosine similarity instead of dot product in neural networks. In *Proc. Int. Conf. Artificial Neural Networks (ICANN)*, Lecture Notes in Computer Science, vol. 11139, pp. 382–391, 10.1007/978-3-030-01418-6_38 (Cham, Switzerland: Springer, 2018).

[23] Pedronette, D. C. G., Gonçalves, F. M. F. & Guilherme, I. R. Unsupervised manifold learning through reciprocal NN graph and connected components for image retrieval tasks. *Pattern Recognition* **75**, 161–174. 10.1016/j.patcog.2017.05.009 (2018).

[24] Jiang, J., Wei, Y., Feng, Y., Cao, J. & Gao, Y. Dynamic hypergraph neural networks. In *Proc. 28th Int. Joint Conf. Artificial Intelligence (IJCAI)*, pp. 2635–2641 10.24963/ijcai.2019/366 (2019).

[25] Beyer, K., Goldstein, J., Ramakrishnan, R. & Shaft, U. When is ‘nearest neighbor’ meaningful? In *Proc. Int. Conf. Database Theory (ICDT)*, Lecture Notes in Computer Science, vol. 1540, pp. 217–235, 10.1007/3-540-49257-7_15. (Berlin, Heidelberg: Springer, 1999).

[26] Brugada, J. et al. 2019 ESC guidelines for the management of patients with supraventricular tachycardia. *Eur. Heart J.* **41**(5), 655–720. 10.1093/eurheartj/ehz467 (2020). 31504425 10.1093/eurheartj/ehz467

[27] Al-Khatib, S. M. et al. 2017 AHA/ACC/HRS guideline for management of patients with ventricular arrhythmias and the prevention of sudden cardiac death: Executive summary: A report of the American College of Cardiology/American Heart Association Task Force on Clinical Practice Guidelines and the Heart Rhythm Society. *Heart Rhythm* **15**(10), e190–e252. 10.1016/j.hrthm.2017.10.035 (2018). 29097320 10.1016/j.hrthm.2017.10.035

[28] Wang, Q., Huang, J., Shen, T. & Gu, Y. EHGNN: Enhanced hypergraph neural network for hyperspectral image classification. *IEEE Geosci. Remote Sens. Lett.* **21**, 5504405 (2024).

[29] Li, K., Huang, Z. & Jia, Z. RAHG: Role-aware hypergraph neural network for node classification. *IEEE Trans. Netw. Sci. Eng.* **10**, 2098–2108 (2023).

[30] Kiranyaz, S., Avci, O., Abdeljaber, O., Ince, T., Gabbouj, M. & Inman, D. J. “1D convolutional neural networks and applications: A survey,” *arXiv preprint *arXiv:1905.03554, (2019). [Online]. Available: https://arxiv.org/abs/1905.03554

[31] Fan, J. et al. An enhanced residual learning framework for graph neural networks based on dual random walk. *Knowl. Based Syst.* **324**, 113822. 10.1016/j.knosys.2025.113822 (2025).

[32] Wen, X., Yu, X., Wang, Y., Yang, C. & Sun, Y. A hybrid 3D–2D feature hierarchy CNN with focal loss for hyperspectral image classification. *Remote Sensing* **15**, 4439. 10.3390/rs15184439 (2023).

[33] Hannun, A. Y. et al. Cardiologist-level arrhythmia detection using deep neural networks. *Nat. Med.* **25**, 65–69 (2019). 30617320 10.1038/s41591-018-0268-3 PMC6784839

[34] Kachuee, M., Fazeli, S. & Sarrafzadeh, M. ECG heartbeat classification: A deep transferable representation. In *2018 IEEE International Conference on Healthcare Informatics (ICHI)* 443–444 (New York City, NY, USA, 2018). 10.1109/ICHI.2018.00092

[35] Teijeiro, T., Félix, P., Presedo, J. & Castro, D. Heartbeat classification using abductive interpretation of ECG. *IEEE J. Biomed. Health Inform.* **22**, 409–420 (2018). 27893401 10.1109/JBHI.2016.2631247

[36] Afkhami, R. G., Azarnia, G. & Tinati, M. A. Cardiac arrhythmia classification using statistical and mixture modeling features of ECG signals. *Pattern Recogn. Lett.* **70**, 45–51 (2016).

[37] Hammad, M. et al. A multitier deep learning model for arrhythmia detection. *IEEE Trans. Instrum. Meas.* **70**, 3022917 (2021).

[38] Mousavi, S., Afghah, F., Khadem, F. & Acharya, U. R. ECG language processing (ELP): A new technique to analyze ECG signals. *Comput. Methods Programs Biomed.* **202**, 105959 (2021). 33607552 10.1016/j.cmpb.2021.105959 PMC8009849

[39] Xu, X., Jeong, S. & Li, J. Interpretation of ECG rhythm by combined CNN and BiLSTM. *IEEE Access* **8**, 125380–125388 (2020).

[40] Xia, Y., Xiong, Y. & Wang, K. A transformer blended with CNN and denoising autoencoder for arrhythmia classification. *Biomed. Signal Process. Control* **86**, 105271 (2023).

[41] Zhao, X., Liu, Z., Han, L. & Peng, S. *ECGNN: Enhancing abnormal recognition in 12-lead ECG with graph neural network*. In *2022 IEEE International Conference on Bioinformatics and Biomedicine (BIBM)* 1411–1416 (Las Vegas, NV, USA, 2022). 10.1109/BIBM55620.2022.9995419

[42] Wang, D., Ao, B., Gao, X. & Xie, Y. A study on ECG classification based on IIVYA-SE-CNN-BiLSTM. In Proc.,. Int. Conf. Artificial Intelligence, Human-Computer Interaction and Robotics (AIHCIR). *Hong Kong, China* **2024**, 274–278. 10.1109/AIHCIR65563.2024.00053 (2024).

[43] Li, Z.-X., Hsu, Y.-S., Chou, P.-Y. & Lin, C.-H. ECG signal classification using 1D ResNet-18 with integrated CBAM and auxiliary classifier, in Proc.,. 1st Int. Conf. Consumer Technology (ICCT-Pacific). *Matsue, Japan* **2025**, 1–4. 10.1109/ICCT-Pacific63901.2025.11012832 (2025).

[44] Lim, J., Han, D., Nejad, M. P. S. & Chon, K. H. ECG classification via integration of adaptive beat segmentation and relative heart rate with deep learning networks. *Comput. Biol. Med.* **181**, 109062. 10.1016/j.compbiomed.2024.109062 (2024). 39205344 10.1016/j.compbiomed.2024.109062 PMC12070331

[45] Rahul, J., Marpe, S., Sharma, L. D. & Bohat, V. K. An improved cardiac arrhythmia classification using an RR interval-based approach. *Biocybern. Biomed. Eng.* **41**(2), 656–666. 10.1016/j.bbe.2021.04.004 (2021).

[46] Saputra, D. C. E., Sunat, K. & Ratnaningsih, T. SMOTE-MRS: A novel SMOTE-multiresolution sampling technique for imbalanced distribution to improve prediction of anemia. *IEEE Access* **12**, 154675–154699. 10.1109/ACCESS.2024.3482968 (2024).

[47] Yadav, A. K., Alam, B. & Pal, O. Evaluating SMOTE-ENN impact on EEG signal seizure detection and classification. *Procedia Comput. Sci.* **258**, 2920–2929. 10.1016/j.procs.2025.04.552 (2025).

[48] Kelesis, D., Fotakis, D. & Paliouras, G. Analyzing the effect of residual connections to oversmoothing in graph neural networks. *Mach. Learn.* **114**, 184. 10.1007/s10994-025-06822-0 (2025).

[49] Xu, Y. & Cao, S. Building engineering cost prediction model based on TSNE and improved grey correlation algorithm. *Procedia Comput. Sci.* **228**, 957–965. 10.1016/j.procs.2023.11.126 (2023).

[50] Swain, C. & Swain, J. Data analysis and visualization tools (Reference Module in Chemistry, Molecular Sciences and Chemical Engineering. *Elsevier*10.1016/B978-0-443-29808-0.00007-8 (2025) (**ISBN 9780124095472**).

